# Platelet disturbances correlate with endothelial cell activation in uncomplicated *Plasmodium vivax* malaria

**DOI:** 10.1371/journal.pntd.0007656

**Published:** 2020-07-20

**Authors:** João Conrado Khouri Dos-Santos, João Luiz Silva-Filho, Carla C. Judice, Ana Carolina Andrade Vitor Kayano, Júlio Aliberti, Ricardo Khouri, Diógenes S. de Lima, Helder Nakaya, Marcus Vinicius Guimarães Lacerda, Erich Vinicius De Paula, Stefanie Costa Pinto Lopes, Fabio Trindade Maranhão Costa

**Affiliations:** 1 Laboratório de Doenças Tropicais–Prof. Luiz Jacintho da Silva. Instituto de Biologia, Universidade Estadual de Campinas, Campinas, Brazil; 2 Pós-graduação em Fisiopatologia Médica, Faculdade de Ciências Médicas, Universidade Estadual de Campinas, Campinas, Brazil; 3 National Institute of Allergy and Infectious Diseases, Bethesda, Maryland, United States of America; 4 Instituto Gonçalo Moniz, Fiocruz Bahia, Salvador, Brazil; 5 Department of Clinical and Toxicological Analyses, School of Pharmaceutical Sciences, University of Sao Paulo, São Paulo, Brazil; 6 Fundação de Medicina Tropical Dr. Heitor Vieira Dourado, Manaus, Brazil; 7 Instituto Leônidas & Maria Deane, Fiocruz Amazônia, Manaus, Brazil; 8 Centro de Hematologia e Hemoterapia–Hemocentro, Universidade Estadual de Campinas, Campinas, Brazil; Faculty of Medicine of University of Colombo, SRI LANKA

## Abstract

Platelets drive endothelial cell activation in many diseases. However, if this occurs in *Plasmodium vivax* malaria is unclear. As platelets have been reported to be activated and to play a role in inflammatory response during malaria, we hypothesized that this would correlate with endothelial alterations during acute illness. We performed platelet flow cytometry of PAC-1 and P-selectin. We measured platelet markers (CXCL4, CD40L, P-selectin, Thrombopoietin, IL-11) and endothelial activation markers (ICAM-1, von Willebrand Factor and E-selectin) in plasma with a multiplex-based assay. The values of each mediator were used to generate heatmaps, K-means clustering and Principal Component analysis. In addition, we determined pair-wise Pearson’s correlation coefficients to generate correlation networks. Platelet counts were reduced, and mean platelet volume increased in malaria patients. The activation of circulating platelets in flow cytometry did not differ between patients and controls. CD40L levels (Median [IQ]: 517 [406–651] vs. 1029 [732–1267] pg/mL, *P* = 0.0001) were significantly higher in patients, while P-selectin and CXCL4 showed a nonsignificant trend towards higher levels in patients. The network correlation approach demonstrated the correlation between markers of platelet and endothelial activation, and the heatmaps revealed a distinct pattern of activation in two subsets of *P*. *vivax* patients when compared to controls. Although absolute platelet activation was not strong in uncomplicated vivax malaria, markers of platelet activity and production were correlated with higher endothelial cell activation, especially in a specific subset of patients.

## Author summary

Endothelial cell activation is a key process in the pathogenesis of *Plasmodium vivax* malaria. Platelets are classically involved in endothelial cell activation in several diseases, but their role in the context of vivax malaria remains unclear. Thrombocytopenia is the most common hematological disturbance in *P*. *vivax*-infected patients, and platelets have been implicated in parasitemia control. In this work, we studied the activation of platelets in association with endothelial cell activation in vivax malaria. Platelets retrieved from infected peripheral blood were non-activated when analyzed by flow cytometry; however, they displayed higher mean volume and significantly reduced counts. We also found higher levels of circulating factors associated with platelet activation (especially soluble CD40L), thrombopoiesis and endothelial cell activation in infected patients. Further, through pair-wise correlation and clustering analysis, we found a subgroup of patients showing significant associations between markers of platelet and endothelial activation in a pattern different from that of endemic controls. Collectively, our findings indicate a role of platelets in endothelial cell activation in vivax malaria and indicate a heterogeneous host response in the setting of uncomplicated disease, a finding to be further explored in future studies.

## Introduction

Platelets play a significant role in malaria, being involved both in the immune response and the pathogenesis of the disease. Recently, platelets have been shown to kill *Plasmodium* parasites of all species, effectively controlling parasitemia and, possibly, disease progression [1, 2]. However, platelets might also induce a proinflammatory state in malaria [3, 4]. Given this apparent importance of platelets, their activation has been investigated but the results are conflicting. Flow cytometry studies have not found significant activation of circulating platelets [1, 5], while measurement of soluble factors derived from activated platelets have shown both negative and positive results [1, 5–8].

Besides their role in immunity, thrombocytopenia (a platelet count <150,000/μL) is the most common hematological alteration in malaria, and there is no definitive mechanistic explanation to its occurrence [9, 10]. Besides being a marker of the disease, thrombocytopenia has clinical implications. In a retrospective cohort, patients who died from *Plasmodium falciparum* malaria had lower platelet counts in comparison to those with less severe disease [11]. In patients with *P*. *vivax* malaria, platelets counts were significantly lower in those with complications [12] or severe disease [13]. In addition, patients with thrombocytopenia showed higher levels of endothelial cell (EC) activation markers compared to those with normal platelet counts [14]. This association between reduced platelet counts and EC activation is of special importance giving the known cross-talk of these two cell lines in diverse disease settings [15–17], and the relevance of EC activation in malaria pathogenesis.

EC activation is present in all malaria species, occurring in both mild and severe cases [18–20]. In terms of pathogenesis, EC activation is important for *P*. *falciparum*-infected erythrocytes adhesion to microvasculature, avoiding immunological clearance and leading to severe disease, while inducing more EC damage [21, 22]. *P*. *vivax*-infected erythrocytes also adhere to EC [23, 24], but the magnitude of the phenomenon is smaller, and if this has a role in endothelium pathology and disease severity is not clear [13]. Nonetheless, patients with severe disease present with higher markers of endothelial activation and dysfunction compared both to controls and uncomplicated cases [13, 25, 26], indicating the fundamental role of this event in disease progression.

There is limited information regarding the interplay of platelets and EC in human malaria, with most insights coming from animal models and *in vitro* studies. Importantly, in pediatric cerebral malaria, the most severe presentation of the disease, platelets have been shown to accumulate in the brain microvessels of affected children [3]. Experimental studies have shown that the adhesion of platelets to mice brain endothelial cells was crucial for the development of the syndrome [27], and that platelets contribute to *P*. *falciparum* adhesion to and activation of cultured EC [28, 29].

Therefore, while platelets are implicated in endothelial pathology in diverse disease settings, including malaria due to other *Plasmodium* species, whether they play a role in EC activation during *P*. *vivax* malaria remains to be investigated. In this study, we show that platelet counts were reduced in *P*. *vivax* malaria patients, while circulating markers of platelet activation showed a trend towards elevation. Importantly, platelet activation markers correlated with those related to endothelial activation, indicating a role for platelets in EC pathology in this disease.

## Methods

### Ethics Statement

All subjects enrolled in the study were adults, and samples were taken only after signing of informed consent. The study was approved by the local Research Ethics Committee at Fundação de Medicina Tropical Dr. Heitor Vieira Dourado (FMT-HVD, Manaus, Brazil), under #CAAE: 54234216.1.0000.0005. Seventy-nine patients with *P*. *vivax* malaria, as diagnosed by light microscopy, seen at FMT-HVD and 34 healthy controls were enrolled. All patients included were outpatients that did not meet World Health Organization (WHO) criteria for severe malaria. We further confirmed diagnosis through qPCR assays for both *P*. *vivax* and *P*. *falciparum*. We used previously published nucleotides sequences [30] (S1 Table).

Exclusion criteria: under 18 years of age; pregnancy; in use of antimalarials; chronic disease; medication known to interfere with platelet count/function; smoking. After signing the informed consent, 20 mL of venous blood were drawn by venipuncture in a syringe with 15% acid citrate dextrose as anticoagulant to minimize *in vitro* platelet activation. Complete blood counts were done within 15 minutes of blood sampling with a Sysmex KX21N counter.

### Platelet isolation and poor platelet plasma preparation

Whole blood was centrifuged at 180 g for 18 minutes at room temperature, without brake for gradient formation, to obtain the platelet rich plasma (PRP). The PRP was centrifuged at 100 g for 10 minutes for removal of residual leukocytes, and subsequently centrifuged at 800g for 20 minutes to obtain the platelet pellet. Prostaglandin E1 300 nM was used to minimize platelet aggregation. The supernatant of this centrifugation was centrifuged at 1000 g for 10 minutes to obtain platelet poor plasma (PPP).

### Platelet parameters

Within 15 minutes of sampling, complete blood counts were performed. Platelet activation was assessed in PRP using anti-CD61 antibody (PerCp-Cy5.5), PAC-1 (FITC) and anti-P-selectin (PE) antibodies, by flow cytometry (FACSCanto, BD) and analysis with FlowJo software (Free Star). The same panel was used to assess whether the incubation of PPP (50% v/v for 10 min at 37°C) from malaria patients was capable to activate platelets from a healthy donor. For this assay specifically, we used mean fluorescence intensity to compare the groups.

We also measured circulating factors associated with platelet activation and production in the patients’ plasma, using a multiplex-based cytokine assay (R&D Systems): sCD40L, P-selectin, CXCL4 and thrombopoietin (TPO), IL-11, as well as circulating markers of EC activation (ICAM-1, E-selectin, von Willebrand Factor (vWF)). We selected 31 patients for the multiplex assay to represent a wide range of parasitemias (260 to 25,150 *Pv*-IE/μL). This subgroup did not differ from the overall population of patients regarding severity of disease, sex proportion, platelet counts and mean parasitemia (the mean age of patients included was lower than that of those not included, but this difference was not significative when considering the population of patients as a whole–S2 Table). We selected nine controls matched for age and sex.

### Network and clustering analysis

The values of each circulating factor measured in the plasma samples, hematological parameters and parasitemia from endemic controls and *P*. *vivax* malaria patients were input in the R software (v 3.4.3) to generate heatmaps and to perform K-means clustering. After running the algorithms, individuals were clustered according to the levels of expression of the mediators in 3 groups, which were named Control, Vivax^low^ and Vivax^high^. The heatmap represents z-scores obtained by centering variables (parasitemia, platelet parameters and markers of endothelial cell activation) with the Scale() Function in R, followed by hierarchical and k-means clustering analysis. In addition, the same software was used to determine pair-wise Pearson’s correlation coefficients to generate correlation networks and the p value to test for non-correlation was evaluated using p ≤ 0.05 as a cut-off. In order to analyze the structure of the networks, the graphics for the network analysis were customized in the Cytoscape software (v 3.5.1) using the prefuse force-directed layout, which in the equilibrium state for the system of forces, determined by the correlation strength, the edges tend to have uniform length, and nodes that are not connected by an edge tend to be drawn further apart.

### Statistical Analysis

Fisher’s exact test was used for categorical data. Student’s t-test was used to compare means between groups with normally distributed data, and data sets with non-normal distributions were compared using the Mann–Whitney test, with p<0.05 considered significant. Data are presented as means and SD unless otherwise stated. Analysis were performed, and the graphs generated in GraphPad Prism5 and R software.

## Results

### Platelet parameters in malaria patients

**Fig 1:**
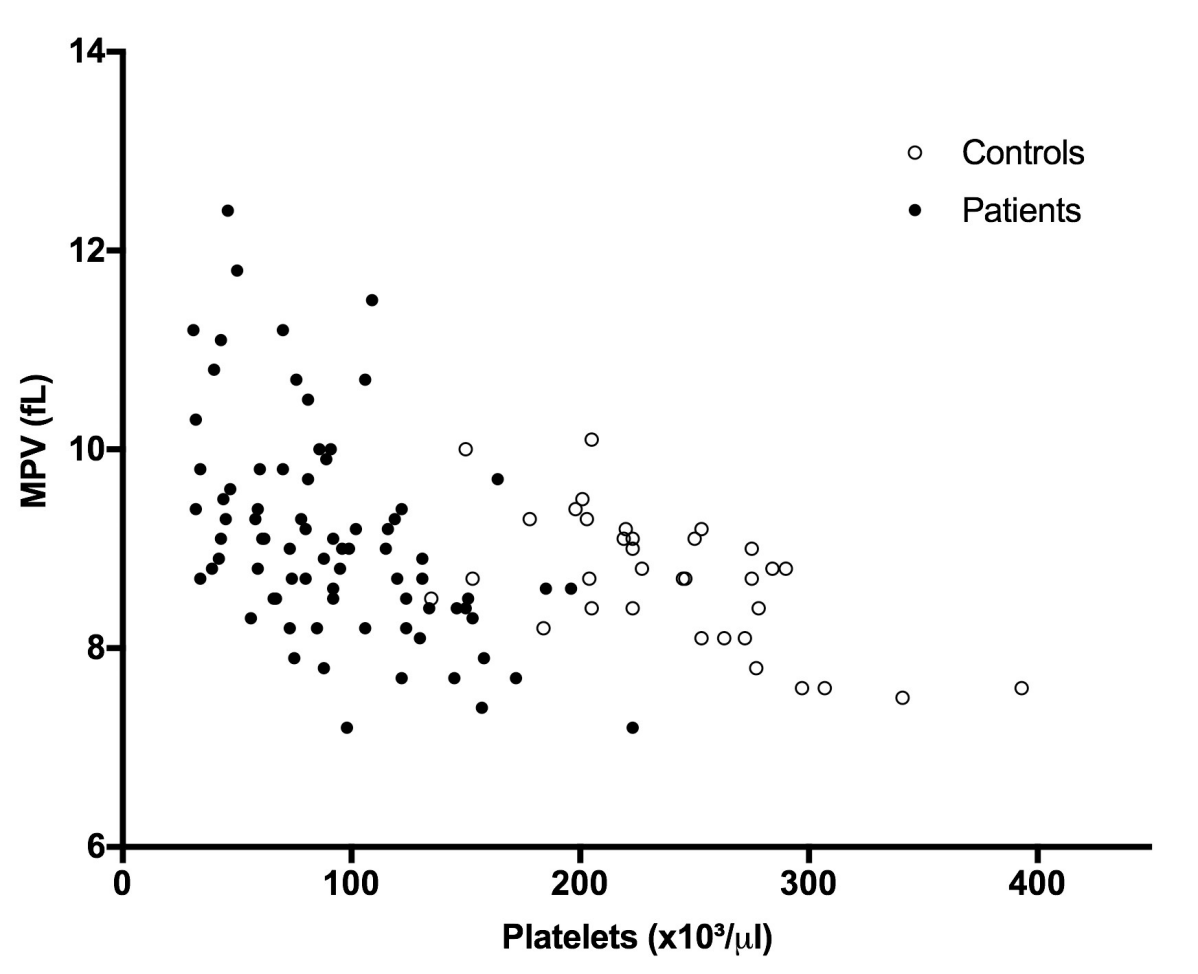
Platelet counts and volume. Platelet count and MPV were inversely correlated in both patients and controls (N: 77 patients and 34 controls). Patients: R = -0.5216, P <0.0001; Controls: R = -0.5808; P <0.001. The blood counter could not determine the MPV for two of the 79 patients.

Platelet counts were significantly reduced in patients (Mean ± SD: 91.6±43.3 x10^9^/L vs. 236.0±54.2 x10^9^/L, P<0.0001), yielding an 87.3% frequency of thrombocytopenia. Mean Platelet Volume (MPV) was increased in patients (9.1±1.1 fL) compared to controls (8.7±0.7 fL, P = 0.0349), and was inversely correlated with platelet counts in both patients and control groups (Patients r = −0.5006, Controls r = −0.6898, both P<0.0001) (Fig 1). Platelet counts were not correlated with parasitemia.

### Platelet Activation in vivax malaria

**Fig 2:**
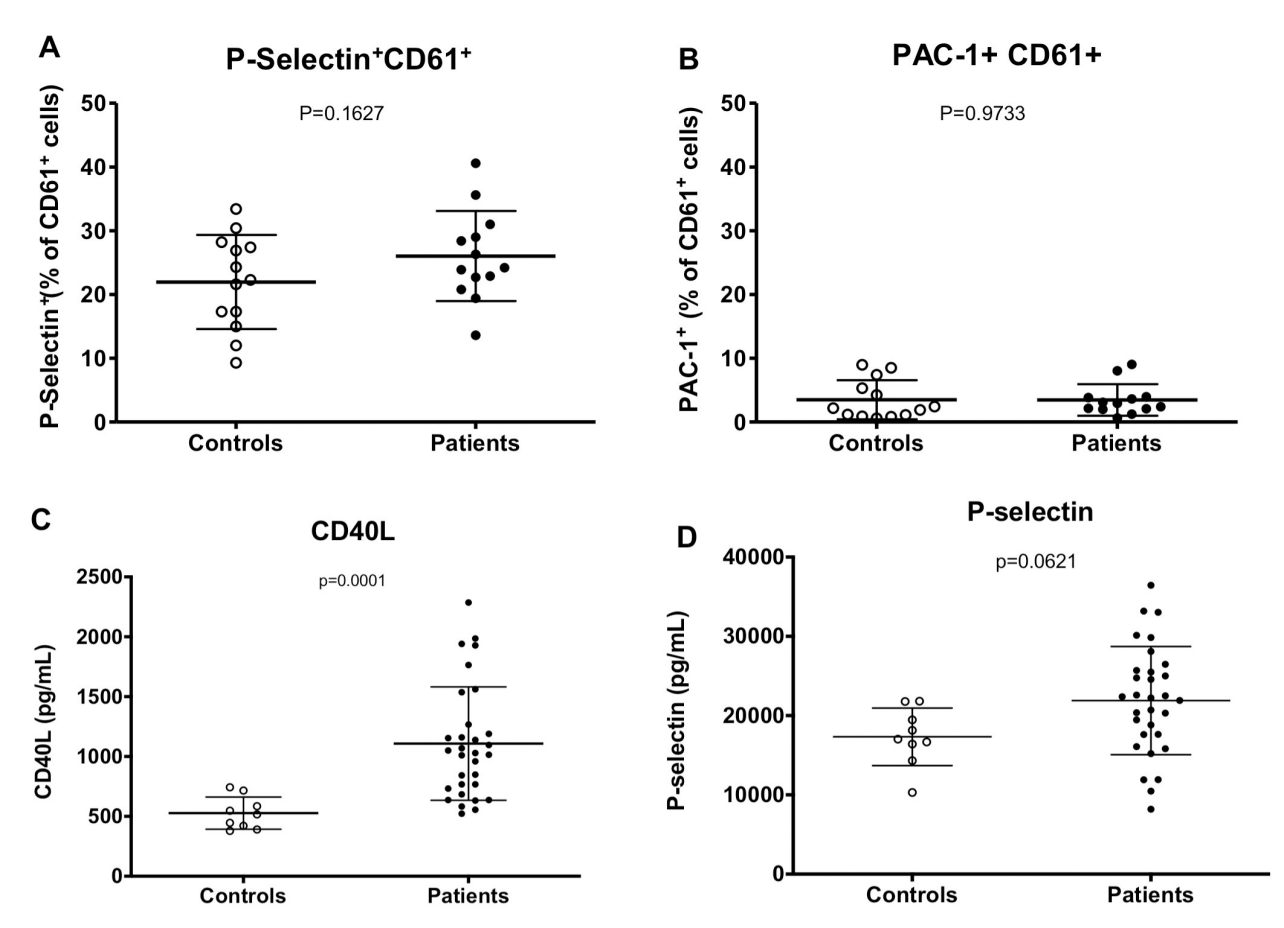
Platelet activation. Percentage of platelet activation by A) anti-P-selectin (N = 14) and B) PAC-1 staining (N = 11). C) Levels of sCD40L. D) Levels of sP-selectin. (Controls, n = 9, Patients n = 31).

Platelet activation is a feature of some thrombocytopenic infections as well of diseases associated with endothelial cell dysfunction [15, 16, 31]. There was no significant difference in the percentage of expression of P-selectin and PAC-1 platelets between patients and controls in flow cytometry (Fig 2A and 2B). Moreover, patients’ PPP failed to activate platelets in comparison to PPP from controls.

In contrast, patients had higher levels of CD40L in plasma (Median [IQ]: 1029 [732–1267] vs. 517 [406–651] pg/mL, *P* = 0.0001; Fig 2C), with a 2-fold increase in comparison to controls. P-selectin showed a trend towards elevated levels in patients (Median [IQ]: 22.2 [17.6–25.7] vs. 17.0 [15.4–20.6] ng/mL, *P* = 0.0621; Fig 2D), while CXCL4 levels (Median [IQ]: 1420 [683–2813] vs. 784 [584–1239] ng/mL, *P* = 0.1236) were not different between the groups (Table 1). Furthermore, when dividing each of these analytes per platelet, a significantly elevated ratio was observed in comparison to controls (S3 Table).

**Table 1:** Plasma levels of markers of platelet activation and production.

Parameter	Controls (n = 9)	Patient (n = 31)	P
	Median [IQ 25–75]		
**CD40L (pg/mL)**	517 [406–651]	1029 [732–1267]	0.0001
**P-Selectin (ng/mL)**	17.0 [15.4–20.6]	22.2 [17.6–25.7]	0.0621
**CXCL4 (pg/mL)**	784 [584–1239]	1420 [683–2813]	0.1236
**TPO (pg/mL)**	2046 [1652–2194]	2996 [2554–3402]	<0.0001
**IL-11 (pg/mL)**	3547 [2904–4298]	5748 [4687–6448]	<0.0001
**ICAM-1 (ng/mL)**	323 [260–464]	634 [456–849]	0.0026
**E-Selectin (ng/mL)**	26.4 [22.5–33.7]	56.7 [41.5–74.1]	0.0001
**VWF (pg/mL)**	126 [120–150]	218 [199–277]	<0.0001

### Thrombopoiesis

Acute reductions in platelet numbers and inflammatory states disturb thrombopoiesis [32, 33]. Therefore, we measured the circulating levels of the cytokines thrombopoietin (TPO) and IL-11, important players in the production of platelets in health and disease. A 50% increase in these two markers was observed in malaria patients (Table 1), and they were significantly correlated (Pearson r = 0.8476, 95%CI: 0.7049–0.9243, P<0.0001). There was no correlation between TPO and platelet counts.

### Endothelial cell activation

In accordance with previous reports [18], EC activation was present in our cohort, with patients displaying higher levels of circulating ICAM-1, E-selectin and vWF (Table 1). Our findings confirm that EC activation occurs even in non-severe vivax malaria cases.

### Correlations and networks

**Fig 3:**
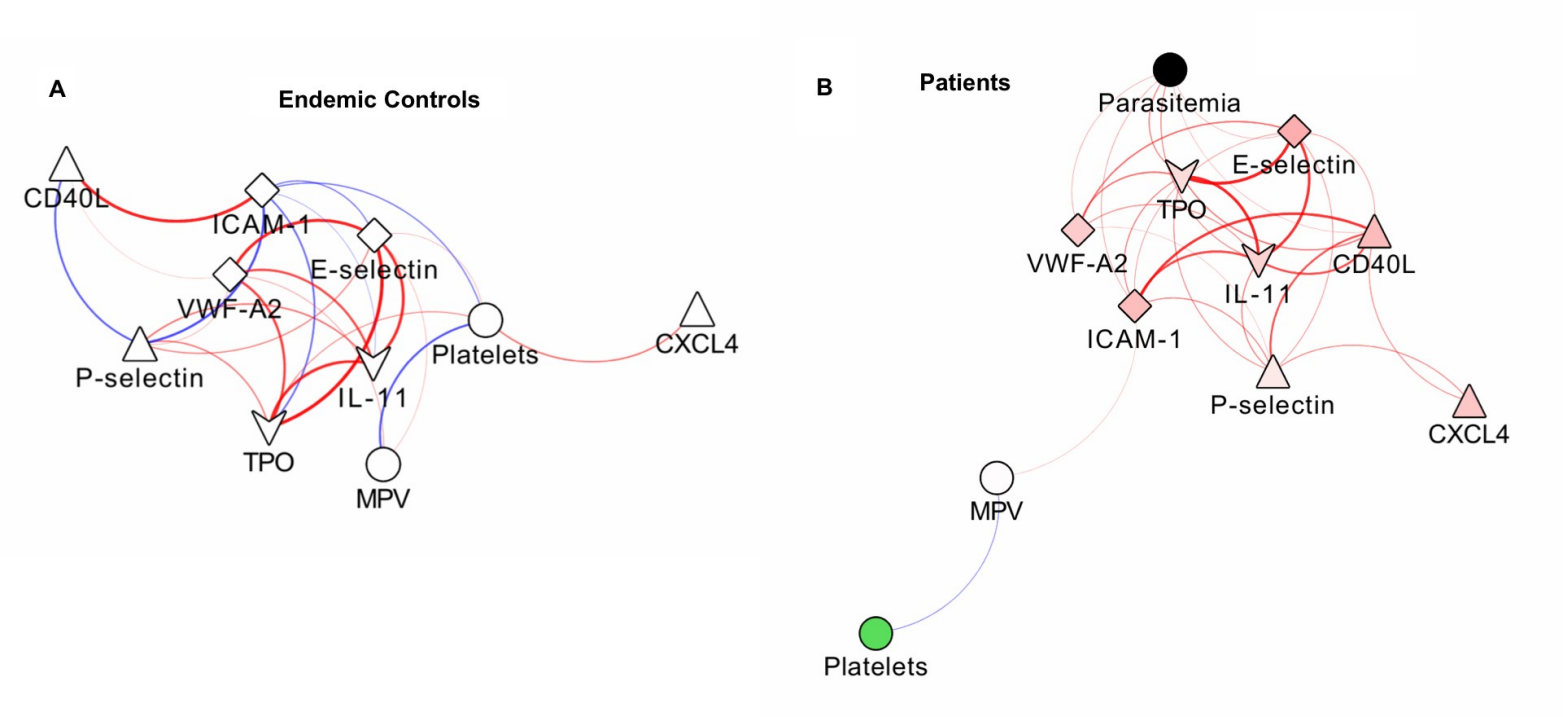
Correlation between markers of platelet and endothelial cell activation. Networks of the correlations between platelet parameters and markers of endothelial cell activation in controls (A) and patients (B), using a prefuse force-directed layout done in Cytoscape software (v 3.5.1). The symbols of the nodes represent biological functions of the molecules presented, triangle represents markers of platelet activation, V shape represents markers of thrombopoiesis, diamond represent endothelial cell activation markers and the circles clinical parameters such as platelets, mean platelet volume and parasitemia. The colors in the nodes represent the fold change in relation to control levels. Because endemic controls do not have parasitemia, the node is represented in black. Each connecting line (edge) represents a significant interaction detected by the network analysis using the R software. Correlation strength is represented by edge color transparency and width. Positive correlations are represented by red edges and negatives correlations are represented by blue edges.

Markers of platelet activation, thrombopoiesis and EC activation were significantly higher in *P*. *vivax* malaria patients in relation to endemic controls. Importantly, all the associations between platelet and EC markers were positive, a change of pattern in relation to controls, in which both negative and positive associations occurred (Fig 3A and 3B). Parasitemia was significantly correlated with the markers of thrombopoiesis TPO and IL-11 and with ICAM-1.

**Fig 4:**
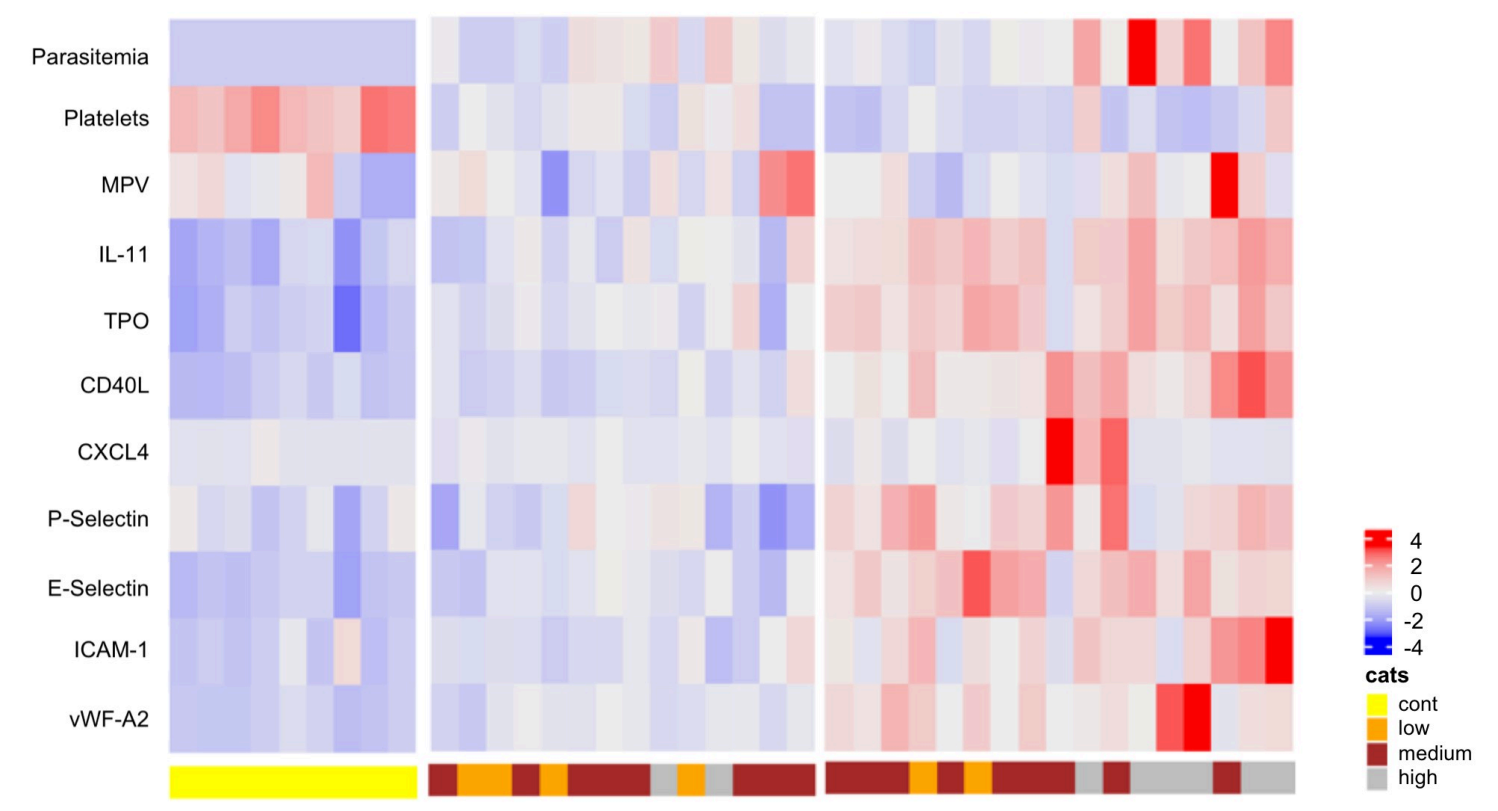
Heatmap of analyte expression. The heatmap represents z-scores obtained by centering variables (parasitemia, platelet parameters and markers of endothelial cell activation) with the Scale() Function in R, followed by hierarchical and k-means clustering analysis, showing three distinct clusters of controls and two groups of patients. Yellow: Controls; Orange: low parasitemia; Brown: moderate parasitemia; Gray: high parasitemia.

**Fig 5:**
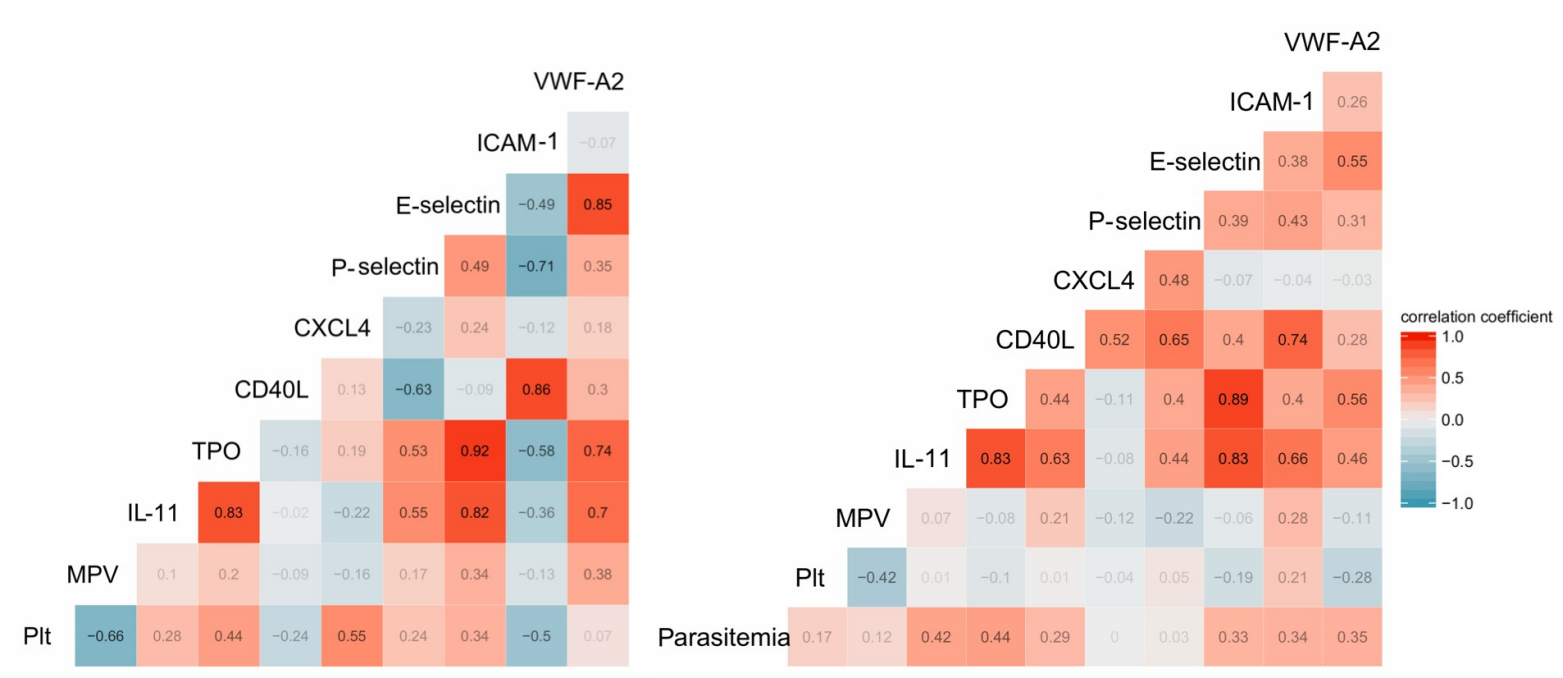
Correlation values. The value for each pair of correlation between the parameters measured in the plasma from controls (A) and patients (B). Plt: platelet count.

Additionally, the heatmap generated from the expression of the analytes revealed a defined separation of controls and two different groups of *P*. *vivax* malaria patients (Fig 4). In comparison to endemic control individuals one group of *P*. *vivax* malaria patients had a lower overall variation in the response. The second group of patients clearly showed a more potent response to the infection, with a higher variation in the expression of platelet activation and EC activation markers. Fig 5A and 5B displays the value of each pair of correlations.

## Discussion

In the current study, we aimed to assess platelet activation and its relationship with endothelial cell activation in *P*. *vivax* malaria. Thrombocytopenia was the most frequent hematological alteration in our cohort and, as previously reported for malaria [9], was correlated with an increase in MPV in a nonlinear fashion. This pattern of correlation [34] between platelet count and MPV has been shown to be associated with thrombocytopenias due to peripheral consumption of platelets, in contrast to those mainly derived from bone marrow failure [35, 36]. The increased MPV in malaria patients could be important during the disease as larger platelets are thought to be more active in comparison to smaller ones, possibly having a role in preventing bleeding [10], and have been implicated in diseases where endothelial activation and dysfunction play a central role [16].

Adding to the investigation of platelet disturbances during the disease, we also measured the levels of TPO and IL-11, two cytokines central to platelet production. Previous reports have demonstrated a high platelet turnover during malaria [37] and increased megakaryocyte numbers in the bone marrow [38]. The observed rise in TPO and IL-11 levels in our cohort indicates that increased platelet production occurs during vivax malaria in response to the reduction in circulating platelet counts. Nonetheless, TPO levels in the plasma of *P*. *vivax* patients presented no correlation with platelet counts, as opposed to what would be expected based on the classical "sponge" model for thrombopoiesis regulation [39]. This model proposes that TPO levels are regulated simply by platelet number: as platelet counts diminish, more TPO circulates freely, resulting in higher stimulation of platelet production in the bone marrow. These results indicate that during *P*. *vivax* malaria, thrombopoiesis might be regulated by additional mechanisms known to stimulate TPO production, such as IL-6 [40] or activation of the Ashwell-Morell receptor [41].

Interestingly, TPO and IL-11 were positively associated with the markers of platelet and EC activation, highlighting the correlation of thrombopoiesis alterations and inflammatory states [16]. Further, as bone marrow EC plays a fundamental role in megakaryocyte development and platelet production [42], we believe that it would be interesting to investigate if and how peripheral blood endothelium lining takes part in thrombopoiesis. Finally, alterations in platelet production in chronic diseases have major implications for platelet function [31, 40]; whether this is the case in acute disorders, like malaria, would be a relevant question for future studies.

Whether a systemic activation of circulating platelets occurs in malaria is still unclear. While a study has reported altered platelet responses after exposure to *P*. *falciparum* infected erythrocytes (P-IE) *in vitro* [43], direct assessment of platelet activation through flow cytometry has rendered negative results [1, 5]. However, evidence of platelet activation *in vivo*, through measurement of circulating factors, have been shown by other groups [6, 7]. Therefore, on the one hand, our flow cytometry results, both in platelets from patients and in platelet stimulation with patients’ plasma, are in line with what has been previously reported in the literature. On the other hand, our results of a trend towards elevated (albeit not significantly) P-selectin and, especially, elevated sCD40L, argue for some grade of systemic platelet activation, leading to platelet degranulation. Of note, the higher biomarker-to-platelet ratio in patients further suggests platelet degranulation. Interestingly, platelets have been shown to release bioactive sCD40L during vaso-oclusive crisis in sickle cell disease, a finding that implicates platelet-derived sCD40L in vascular events [44]. As CD40L is also involved in T-cell activation, we cannot exclude that the latter is a relevant source of the sCD40L found in the patients. However, platelets are considered the major source of sCD40L in circulation [45].

Endothelial cell activation is a major component of malaria pathogenesis [13, 14, 18–20, 22, 23, 25, 26], a phenomenon with an extensive participation of platelets in different disease settings [16]. In contrast to falciparum malaria, in which infected-erythrocyte cytoadhesion is well characterized as a major factor in pathophysiology, it is less clear by what mechanism EC activation could lead to disease progression in vivax malaria. Nonetheless, the relevance of endothelium involvement in vivax malaria pathogenesis is indicated by higher levels of soluble endothelial markers and evidence of vascular dysfunction in severe cases [13, 25, 26]. Of note, as more evidence accumulates for hidden reservoirs of *P*. *vivax* during vertebrate infection [46], especially outside of the vasculature, EC must have a fundamental role in parasite trafficking in and out of circulation. Moreover, major clinical syndromes that lead to death in severe cases, like Acute Respiratory Distress Syndrome [47–49] and Acute Kidney Injury [50], are associated with endothelial pathology [51, 52] and might occur in vivax malaria in the absence of direct parasite involvement.

As platelets are classically associated with endothelial pathology, we searched for patterns of association between platelet factors and markers of endothelial cell activation. Interestingly, our networks revealed the association of soluble CD40L and P-selectin with ICAM-1 and E-selectin, indicating the interplay between these two cell populations in vivax malaria. Indeed, these platelet markers have been implicated in EC activation in different diseases. Incubation of activated platelets with cultured endothelium leads to its activation through CD40L-CD40 interaction [53], and CD40L has been described as a relevant molecule in the pathogenesis of atherosclerosis [45] and sickle cell disease [44]. P-selectin is also recognized as a relevant molecule for other diseases where platelet-EC cross-talk are central [54]. Importantly, both of these markers have been shown to impact platelet interaction with EC in mouse models of malaria [27, 55].

Notably, the heatmaps further confirmed a distinct pattern of platelet and endothelial cell activation in two subsets of patients (Fig 4), with one more markedly distinct from controls. In recent years, it has been increasingly appreciated that patients with seemingly similar clinical presentations have distinct patterns of inflammatory response; exploring these patterns can contribute to better pathophysiological understanding of diseases and even direct specific treatment [56, 57]. Therefore, we believe that the finding of different subgroups of patients can enhance our comprehension of vivax malaria pathogenesis and reinforces the hypothesis that platelet activation and release of granule content plays a role in the endothelium alterations in *P*. *vivax* malaria [6].

This study has several limitations, with the most important being the small number of patients included in the multiplex-based assay, limiting the generalizability of the findings and the statistical analyses. Moreover, while a role for platelets in endothelial activation in malaria is indicated by our results and has biological foundation, the mechanisms behind this association were not elucidated. Finally, although all patients included in the study presented with uncomplicated malaria, we did not follow patients throughout their illness to assess potential clinical implications of our findings.

In this study, we found evidence that markers of platelet activation and production correlate with endothelial cell activation during vivax malaria. Importantly, these markers categorize patients in clearly distinct groups, further indicating that they are indeed intertwined. Collectively, our findings indicate a role of platelets in endothelial cell activation in vivax malaria and indicate a heterogeneous host response in the setting of uncomplicated disease. Therefore, we believe that future studies to identify the mechanisms of how platelets induce EC pathology in relevant models of vivax malaria are warranted.

## Supporting information

S1 Table Primers and probes for qPCR. (PPTX)

S2 Table All patients vs. patients included in the multiplex-based assay. (PPTX)

S3 Table Comparison of the relationship of platelet marker levels to platelet count in patients and controls. (PPTX)
