# Supplementary material for: Platelet disturbances correlate with endothelial cell activation in uncomplicated Plasmodium vivax malaria
Source: PLoS Negl Trop Dis. 2020 Jul 20;14(7):e0007656. doi: 10.1371/journal.pntd.0007656 (PMC7392343; doi:10.1371/journal.pntd.0007656)
Supplement: S1 Table — (PPTX) [file pntd.0007656.s001.pptx]

## Slide 1
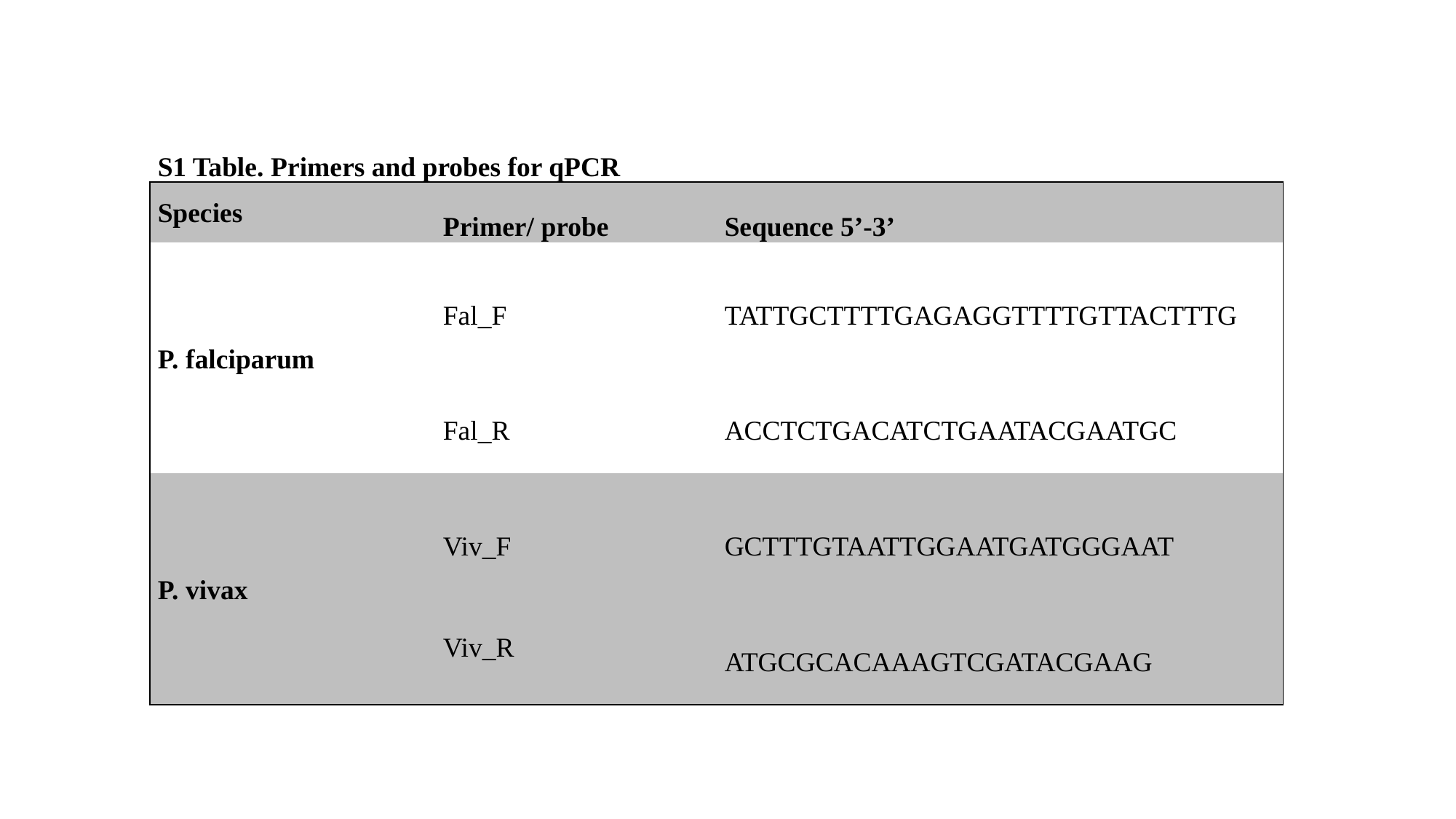

| S1 Table. Primers and probes for qPCR | | |
| --- | --- | --- |
| Species | Primer/ probe | Sequence 5’-3’ |
| P. falciparum | Fal\_F | TATTGCTTTTGAGAGGTTTTGTTACTTTG |
| | Fal\_R | ACCTCTGACATCTGAATACGAATGC |
| P. vivax | Viv\_F | GCTTTGTAATTGGAATGATGGGAAT |
| | Viv\_R | ATGCGCACAAAGTCGATACGAAG |
