# Supplementary material for: Platelet disturbances correlate with endothelial cell activation in uncomplicated Plasmodium vivax malaria
Source: PLoS Negl Trop Dis. 2020 Jul 20;14(7):e0007656. doi: 10.1371/journal.pntd.0007656 (PMC7392343; doi:10.1371/journal.pntd.0007656)
Supplement: S2 Table — (PPTX) [file pntd.0007656.s002.pptx]

## Slide 1
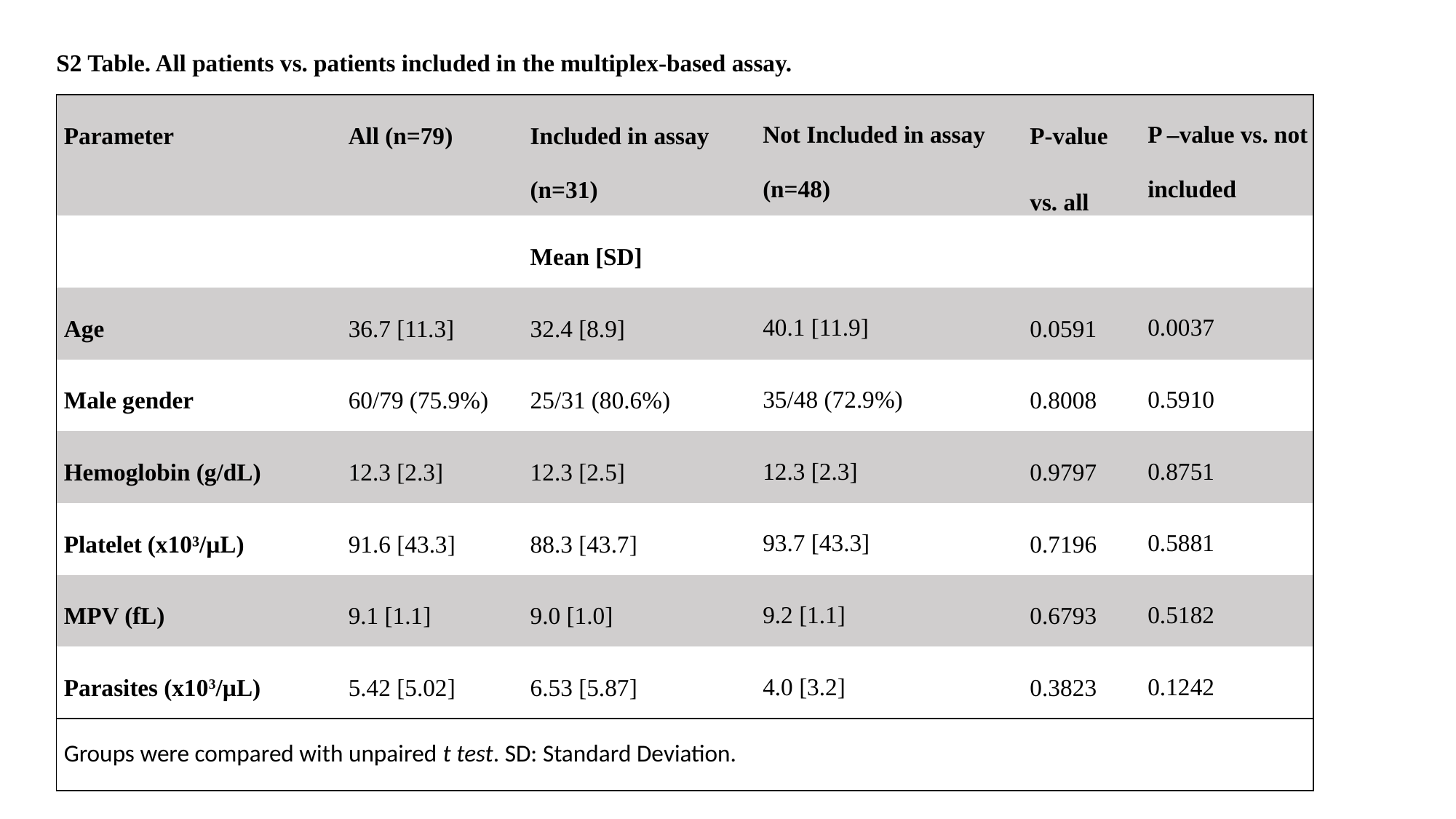

| S2 Table. All patients vs. patients included in the multiplex-based assay. | | | | | |
| --- | --- | --- | --- | --- | --- |
| Parameter | All (n=79) | Included in assay (n=31) | Not Included in assay (n=48) | P-value vs. all | P –value vs. not included |
| | | Mean [SD] | | | |
| Age | 36.7 [11.3] | 32.4 [8.9] | 40.1 [11.9] | 0.0591 | 0.0037 |
| Male gender | 60/79 (75.9%) | 25/31 (80.6%) | 35/48 (72.9%) | 0.8008 | 0.5910 |
| Hemoglobin (g/dL) | 12.3 [2.3] | 12.3 [2.5] | 12.3 [2.3] | 0.9797 | 0.8751 |
| Platelet (x10³/µL) | 91.6 [43.3] | 88.3 [43.7] | 93.7 [43.3] | 0.7196 | 0.5881 |
| MPV (fL) | 9.1 [1.1] | 9.0 [1.0] | 9.2 [1.1] | 0.6793 | 0.5182 |
| Parasites (x103/µL) | 5.42 [5.02] | 6.53 [5.87] | 4.0 [3.2] | 0.3823 | 0.1242 |
| Groups were compared with unpaired t test. SD: Standard Deviation. | | | | | |
