# Supplementary material for: Platelet disturbances correlate with endothelial cell activation in uncomplicated Plasmodium vivax malaria
Source: PLoS Negl Trop Dis. 2020 Jul 20;14(7):e0007656. doi: 10.1371/journal.pntd.0007656 (PMC7392343; doi:10.1371/journal.pntd.0007656)
Supplement: S3 Table — (PPTX) [file pntd.0007656.s003.pptx]

## Slide 1
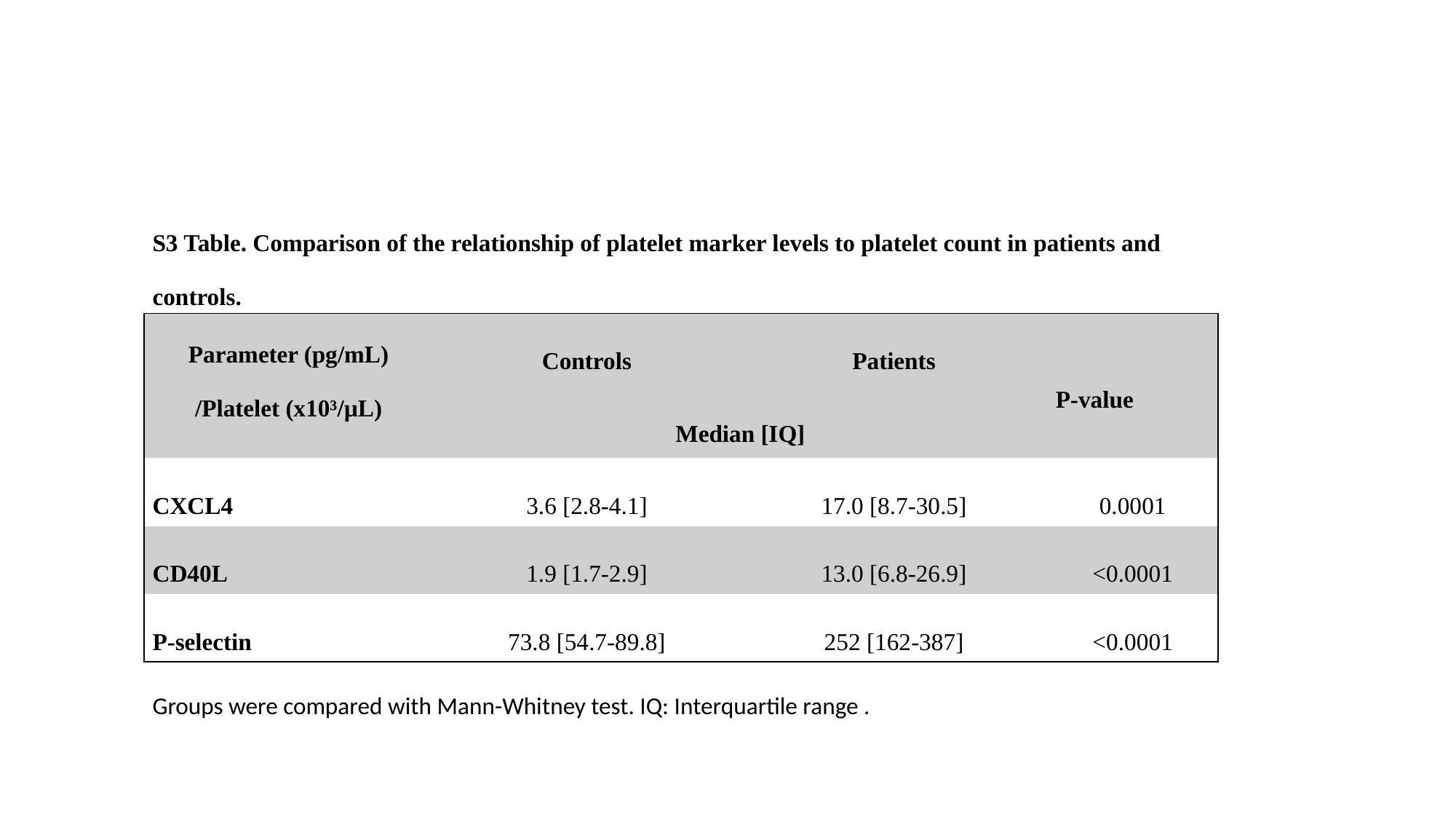

| S3 Table. Comparison of the relationship of platelet marker levels to platelet count in patients and controls. | | | |
| --- | --- | --- | --- |
| Parameter (pg/mL) /Platelet (x10³/µL) | Controls | Patients | P-value |
| | Median [IQ] | | |
| CXCL4 | 3.6 [2.8-4.1] | 17.0 [8.7-30.5] | 0.0001 |
| CD40L | 1.9 [1.7-2.9] | 13.0 [6.8-26.9] | <0.0001 |
| P-selectin | 73.8 [54.7-89.8] | 252 [162-387] | <0.0001 |
| Groups were compared with Mann-Whitney test. IQ: Interquartile range . | | | |
